# Supplementary material for: Occurrence of KPC-71 or the presence of NDM combined with a high expression of blaSHV contributes to low-level cefiderocol resistance in carbapenem-resistant Klebsiella pneumoniae in China
Source: Microbiol Spectr. 2025 Aug 15;13(10):e01593-25. doi: 10.1128/spectrum.01593-25 (PMC12502687; doi:10.1128/spectrum.01593-25)
Supplement: Supplemental figures — Fig. S1 to S7. [file spectrum.01593-25-s0001.docx]

**Figure S1.** Antimicrobial susceptibility of 29 CFDC-resistant CRKP strains.

**
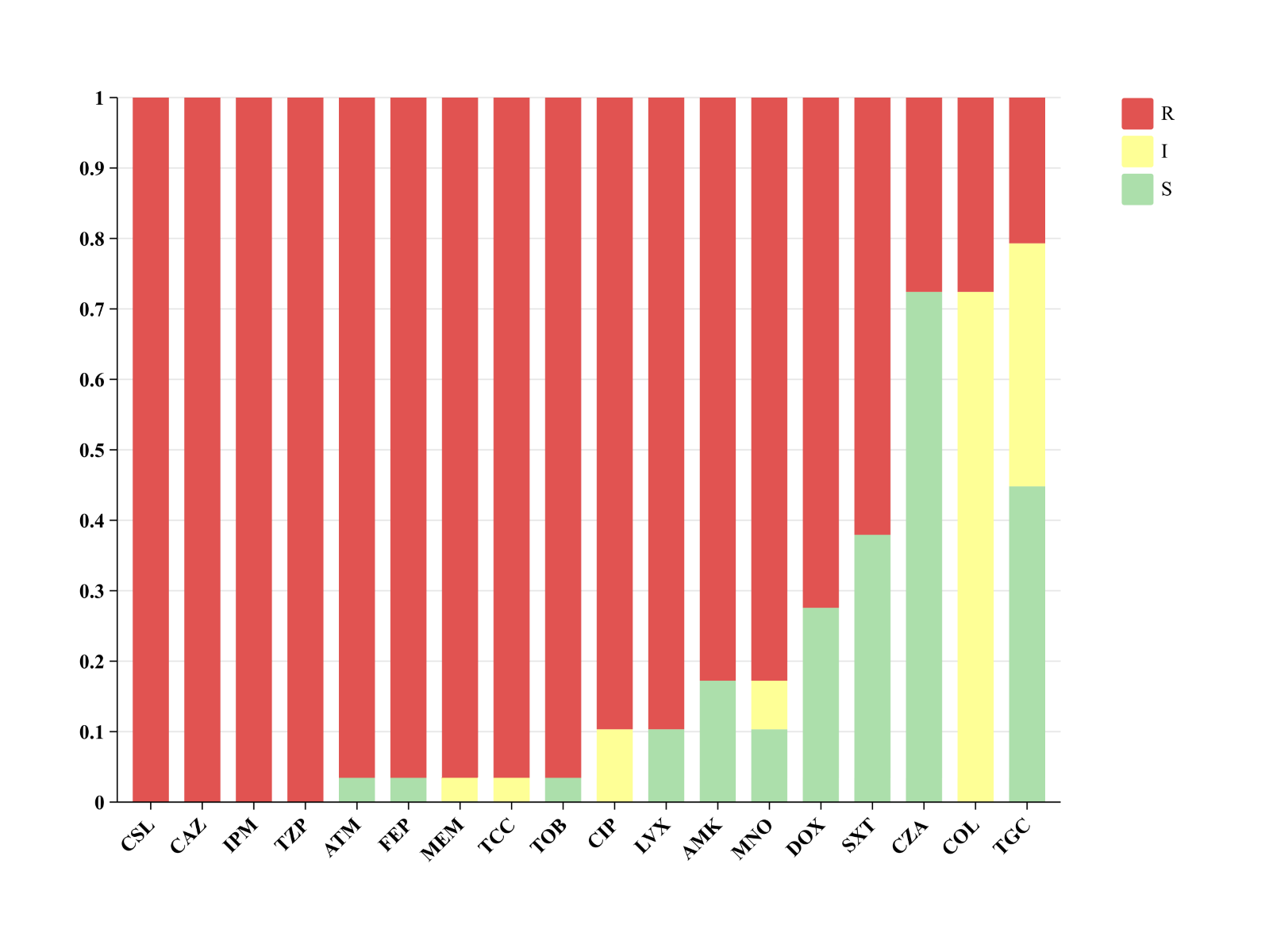
**

**
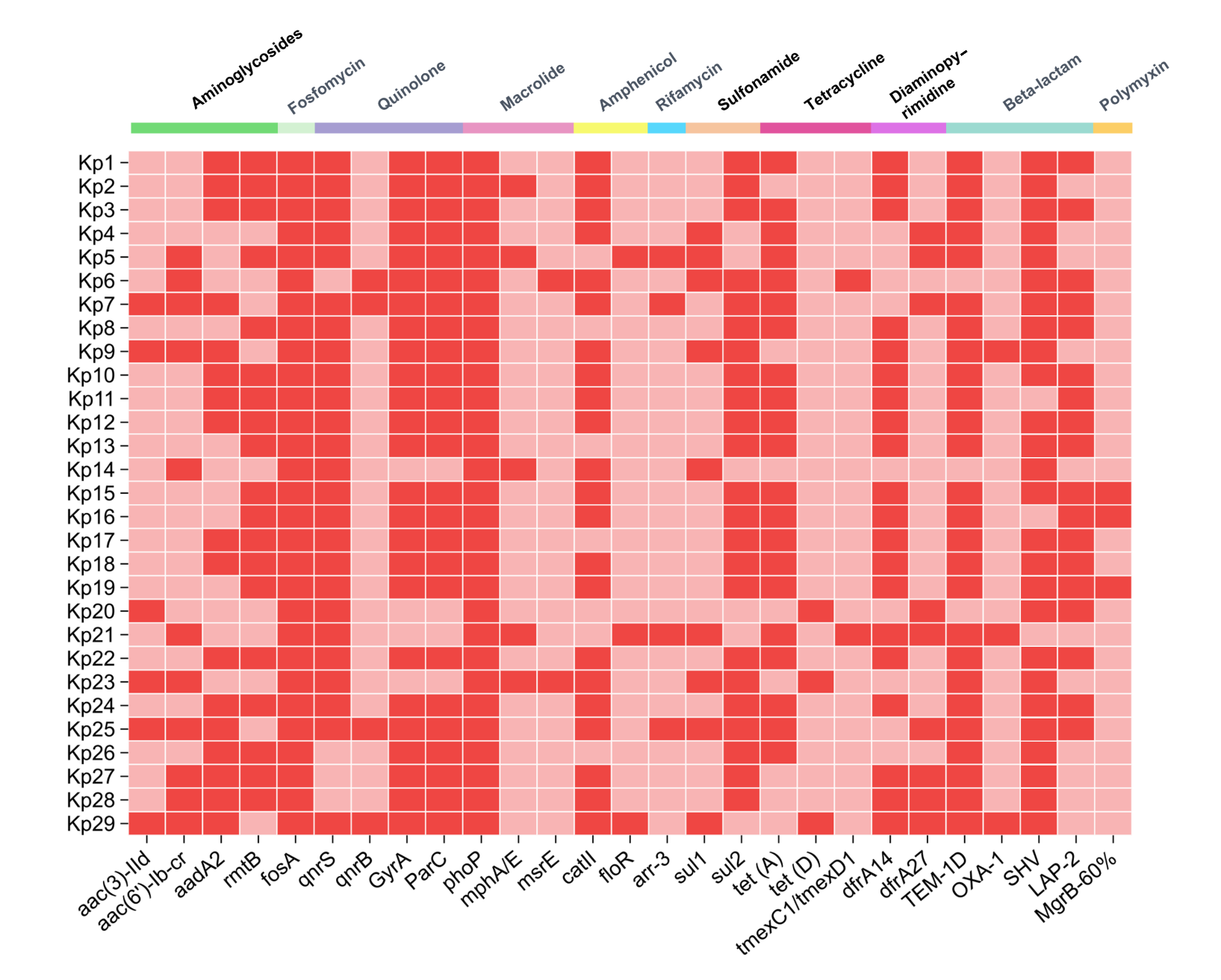
Figure S2.** Heatmap of antibiotic resistance genes of 29 CFDC-resistant strains. The presence of antimicrobial resistance genes in 29 CFDC-resistant CRKP strains was analyzed and visualized using a heatmap. Deep red indicates the presence of a resistance gene, while light red denotes its absence. Resistance genes were categorized based on their associated antibiotic classes.

**Figure S3.** Phylogenetic analysis of 29 CFDC-resistant CRKP strains based on PFGE. PFGE images were imported to Bionumerics software ver. 7.6 (AppliedMaths, Belgium) to identify image bands and construct UPGMA dendrogram. Genetic relatedness was interpreted using a cluster cutoff line set at 80% similarity.

**
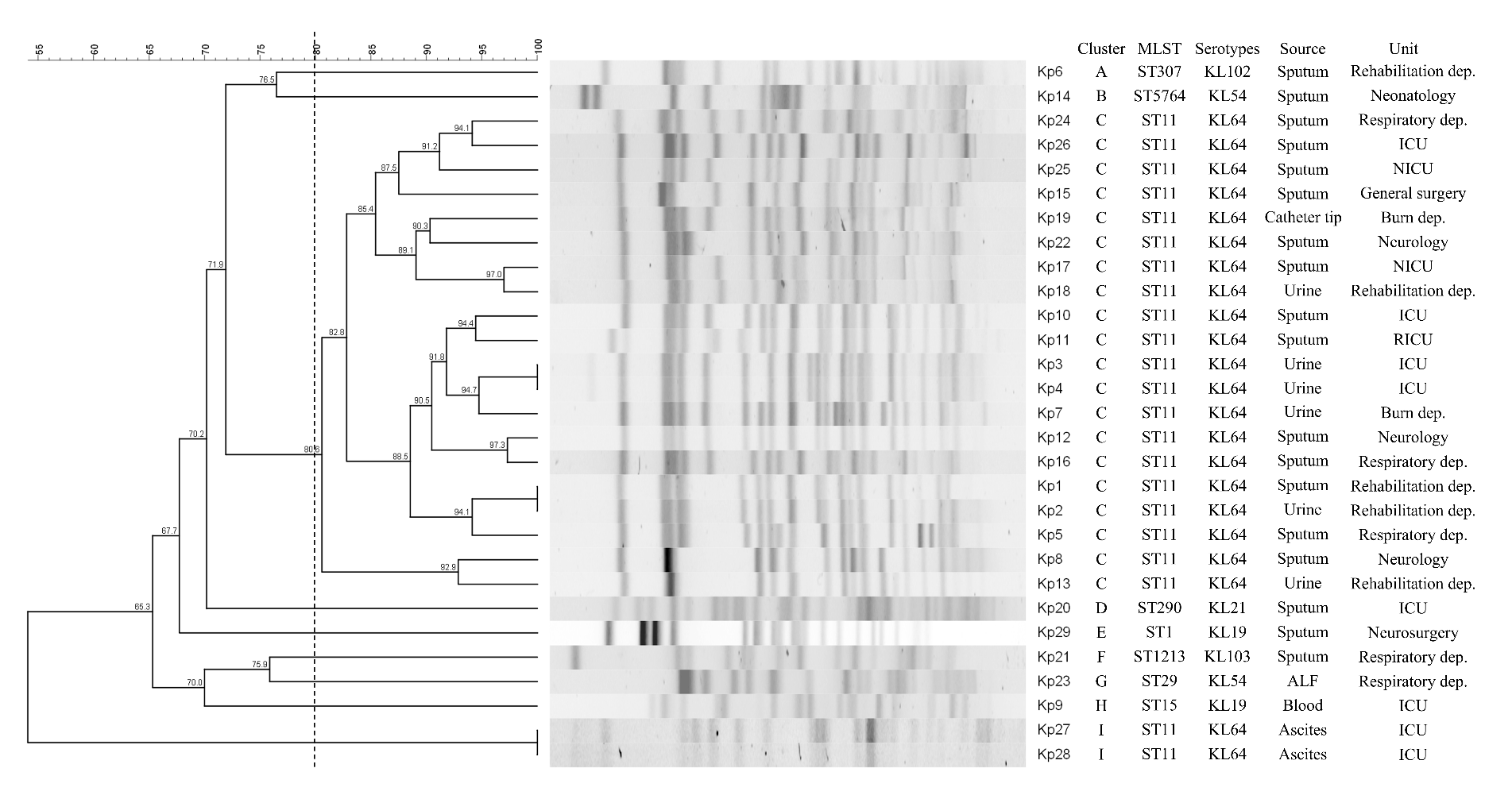
**

**Figure S4.** Comparative genomic context of *bla*_SHV-12_ in Kp22. Upstream (10 kbp) and downstream (10 kbp) sequences flanking the *bla*_SHV-12_ gene on the Kp22 chromosome and plasmid were extracted and aligned. *Bla*_SHV-12_ is coloured in blue, while associated mobile genetic elements are illustrated in cyan. Percent identity between the sequences is indicated by the gradient scale bar. The comparative genomic illustration was generated using Easyfig.


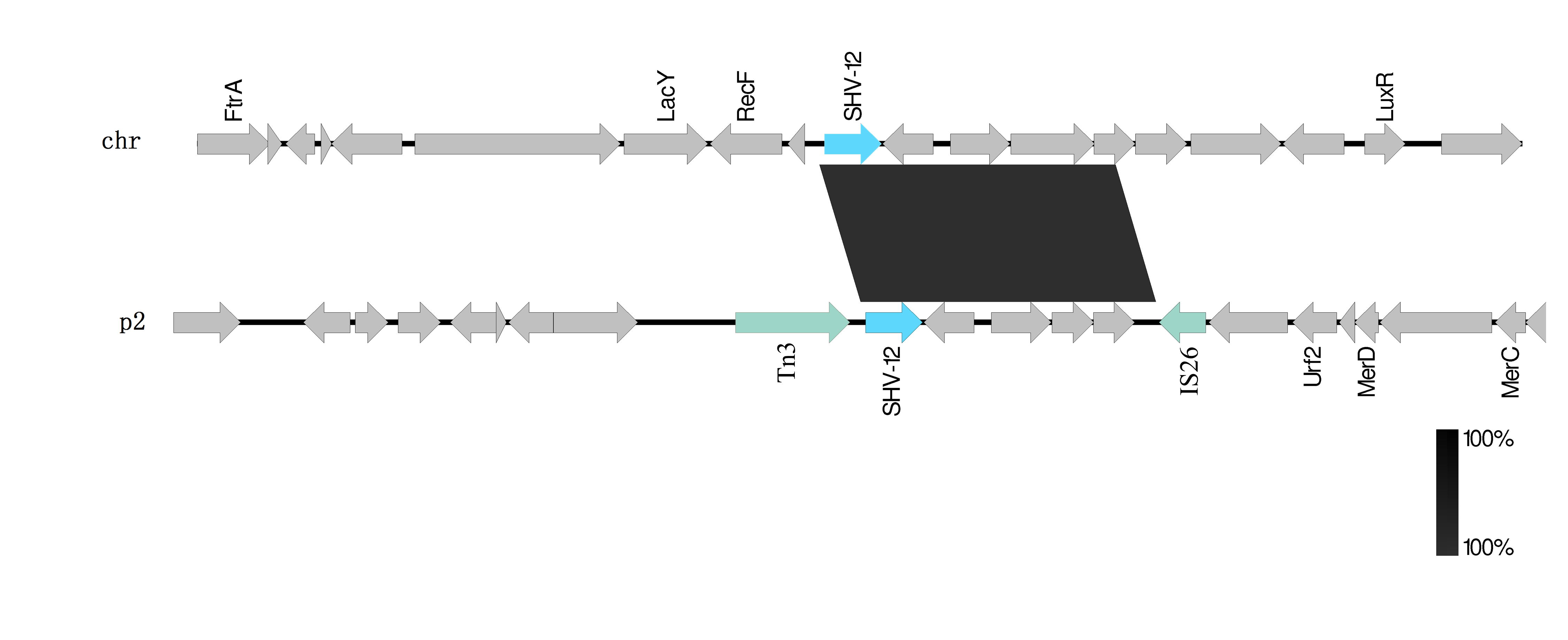


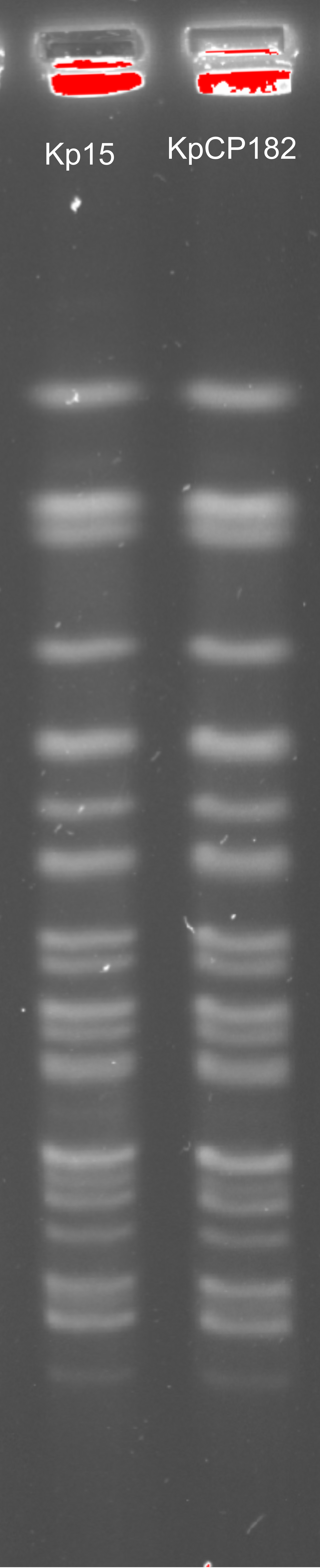
**Figure S5.** PFGE images of Kp15 and KpCp182 isolates from the same patient.

**Figure S6.** Comparative analysis of the genetic structure of KPC plasmids. The innermost layer shows the plasmid pNC75-5, which is most similar to plasmid pKPC from Kp15 based on NCBI BLAST.


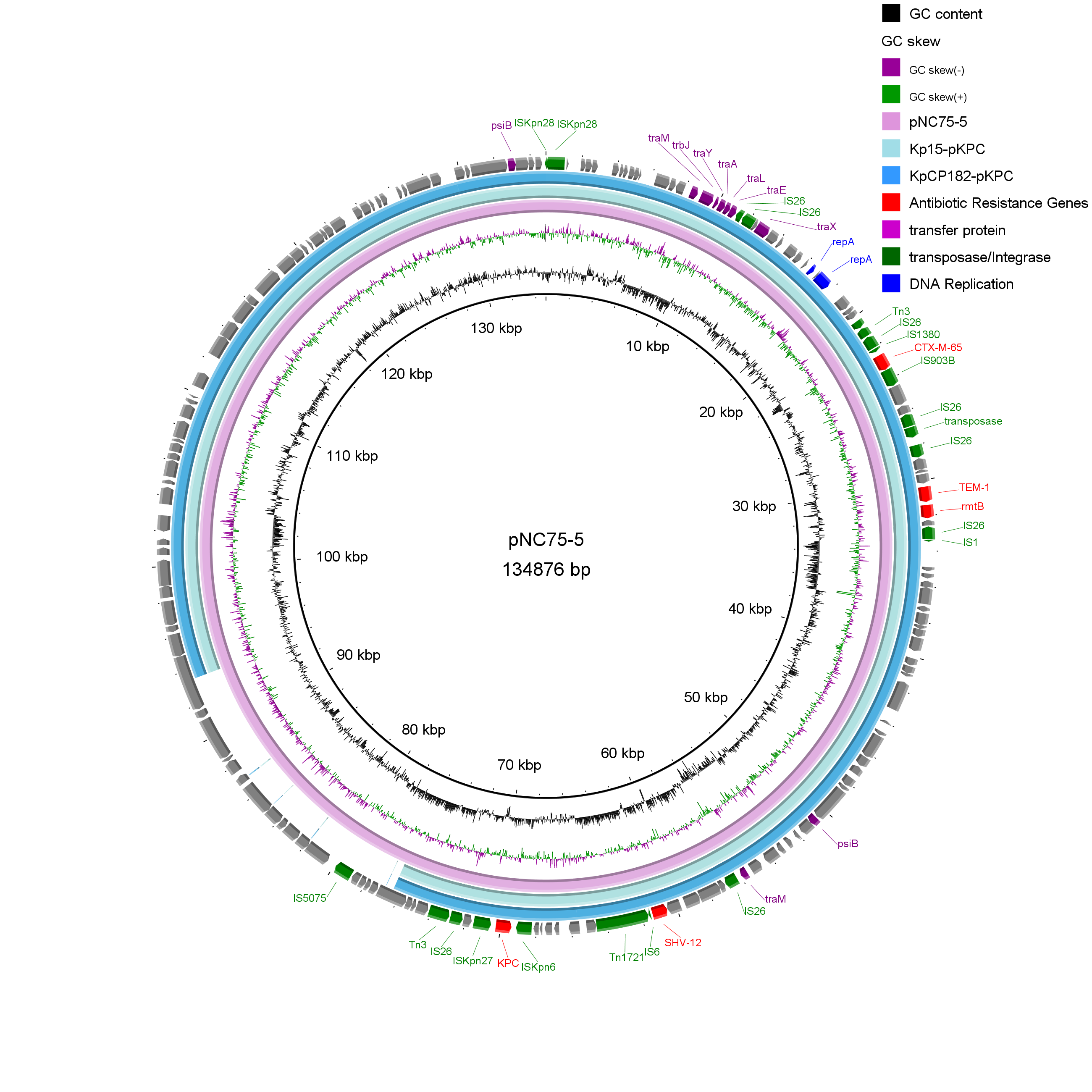


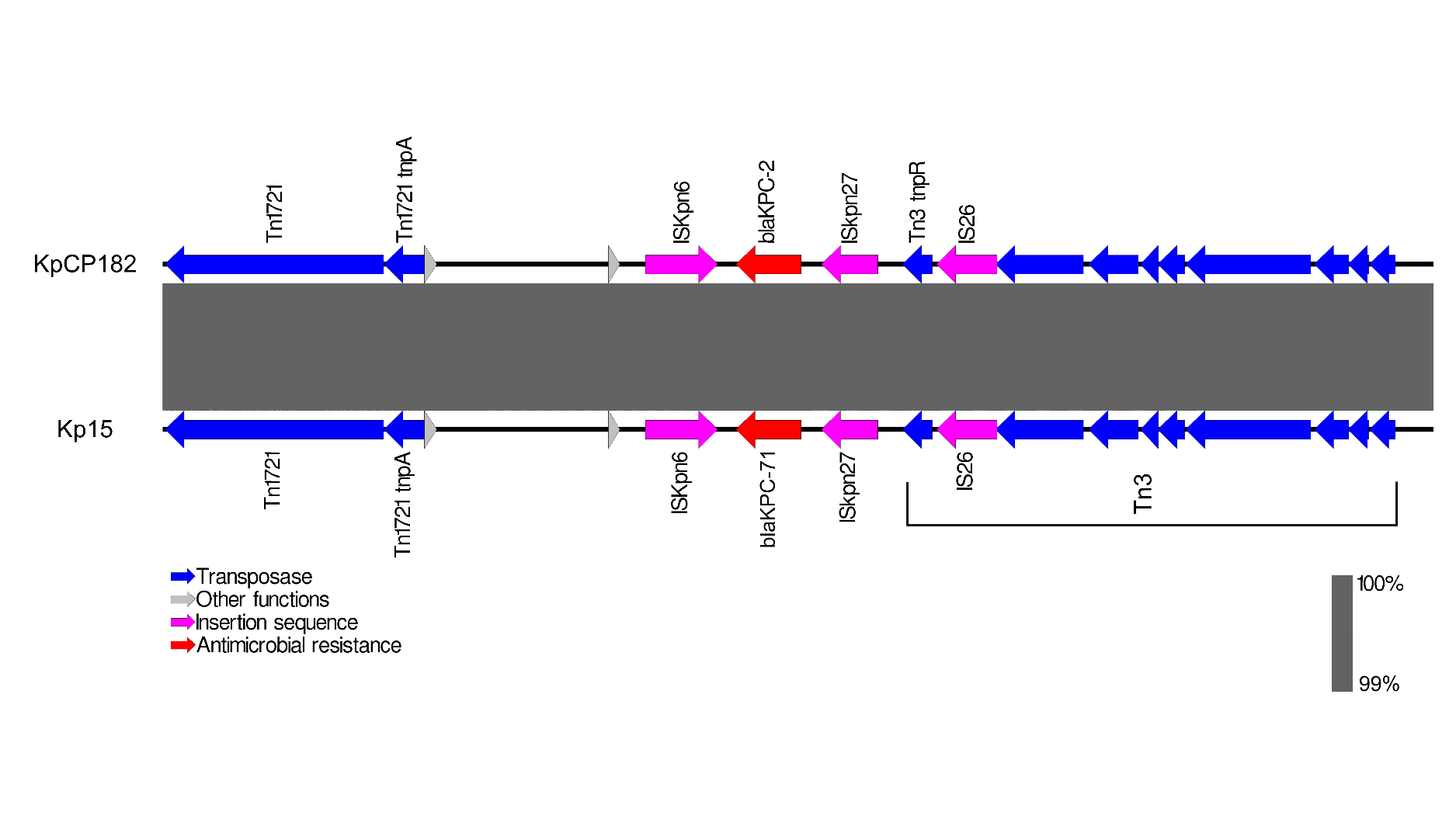
**Figure S7.** The genetic environment surrounding blaKPC-2 and blaKPC-71.
